# Impacts of proactive personality on students' academic achievement: a moderated mediation model

**DOI:** 10.3389/fpsyg.2025.1596032

**Published:** 2025-06-09

**Authors:** Dongmei Liu, Ye Lin, Yongchao Jin, Zongjin Li, Kexin Fu, Rui Yang

**Affiliations:** ^1^School of Science, North China University of Science and Technology, Tangshan, China; ^2^School of Philosophy and Sociology, Jilin University, Changchun, China

**Keywords:** proactive personality, academic achievement, academic self-efficacy, perceived social support, student

## Abstract

**Introduction:**

A proactive personality, characterized by an individual's tendency to take initiative and actively shape their environment, has been increasingly recognized as a critical factor in academic success. Drawing on Job Demands-Resources (JD-R) theory and proactive motivation frameworks, this study investigates the relationship between proactive personality and academic achievement, focusing on the mediating role of academic self-efficacy and the moderating role of perceived social support.

**Method:**

Data on proactive personality, academic self-efficacy, perceived social support, and academic achievement were gathered through WJX, yielding a total of 1,153 completed questionnaires. Statistical analyses were performed using SPSS and Mplus software, including correlation analysis, principal component analysis, *t*-tests, and parametric statistics.

**Results:**

Results indicate that proactive personality positively predicts academic achievement, with academic self-efficacy serving as a significant mediator. Furthermore, perceived social support moderates this relationship, enhancing the positive effects of proactive personality on academic achievement through strengthened self-efficacy.

**Discussion:**

The study highlight the importance of fostering proactive behaviors and providing robust social support systems in educational settings to promote students' academic success.

## 1 Introduction

In the knowledge economy, human capital has emerged as the primary determinant of national competitiveness (Drucker, 1999). As the primary architects of skilled workforces, universities now bear dual responsibilities: cultivating talent capable of sustaining innovation-led growth while demonstrating measurable educational effectiveness. One important criterion for assessing the quality of talent in higher education is students' academic achievement (Tinto, 1993). Consequently, identifying factors that influence academic success and exploring strategies to enhance students' academic achievement have become central foci of scholarly inquiry (York et al., 2015).

In recent years, the influence of personality traits on academic success has garnered increasing attention (Poropat, 2009). Among these traits, a proactive personality—characterized by an individual's propensity to take initiative, shape their environment, and drive positive change—has emerged as a significant predictor of academic performance (Bateman and Crant, 1993). Originally studied in organizational behavior, leadership, and career success contexts (Seibert et al., 2001), proactive personality has been increasingly examined in educational settings. For example, among nursing students, a proactive personality not only enhances perceived employability (Ma et al., 2021) but also optimizes cross-disciplinary learning competencies (Zhu et al., 2024). Proactive students are distinguished by their ability to seek growth opportunities, overcome obstacles, and actively engage in their learning processes (Parker et al., 2010). Such students often exhibit heightened levels of engagement, motivation, and persistence, which are critical determinants of academic achievement (Wang et al., 2016). However, while the direct impact of proactive personality on academic outcomes has been explored, its interplay with other psychological resources remains underexamined. Academic self-efficacy and perceived social support, for instance, are pivotal psychological resources that significantly influence academic performance and mental wellbeing (Kristensen et al., 2023; Hefner and Eisenberg, 2009). Academic self-efficacy fosters intrinsic motivation and resilience, while perceived social support provides extrinsic security and encouragement. Together, these resources enable students to navigate academic challenges and achieve their goals. Understanding the dynamic interplay between these psychological constructs offers valuable insights for designing educational interventions and promoting mental health.

The psychological resources students rely on—self-efficacy and social support—function within a broader theoretical framework. Drawing on the well-established Job Demands-Resources (JD-R) theory (Bakker and Demerouti, 2017), we can analyze academic environments through a similar lens (Bajaba et al., 2021; Tisu et al., 2020). A proactive personality, a critical individual resource, drives self-initiated change and intrinsic motivation, facilitating the acquisition of work resources through enhanced self-efficacy (Sun and Yoon, 2022). Concurrently, social support, a pivotal work resource, is widely recognized for its role in bolstering individuals' capacity to navigate academic stressors and sustain learning motivation (Cohen and Wills, 1985). Against this theoretical backdrop, this study investigates the mechanisms and boundary conditions through which proactive personality influences academic achievement among college students, with academic self-efficacy as a mediating variable and perceived social support as a moderating variable. The findings aim to offer actionable strategies for educators to cultivate supportive learning environments that enhance student engagement and academic success (Zimmerman, 2000).

## 2 Literature review

### 2.1 Proactive personality and students' academic achievement

The conceptual framework of proactive personality has undergone significant theoretical development, with its foundational structure initially established by Bateman and Crant (1993). Building upon this, Frese and his collaborators further defined proactive personality as a stable individual disposition characterized by persistent, self-initiated, goal-directed behavioral patterns. Its defining feature lies in actively shaping the environment rather than passively adapting to it (Frese, 2001). Unlike the traditional Five-Factor Model of personality, proactive personality places particular emphasis on action-oriented behavior, with typical manifestations including actively seeking feedback, persistently tackling complex problems, and driving organizational change (Frese et al., 1997). Frese's research highlights that individuals with high levels of proactive personality not only exhibit stronger intrinsic motivation but, more importantly, can effectively employ error management strategies to transform failures into learning opportunities (Frese and Keith, 2015).

Within Frese's theoretical framework, active behavior is regarded as the concrete behavioral expression of proactive personality, with its core features reflected in planned action and contextual adaptation. Substantial empirical evidence demonstrates that proactive personality, by fostering active behavior, not only significantly enhances individual-level work performance (e.g., task completion efficiency) but also effectively improves organizational-level outcomes such as innovation climate (Frese et al., 2007). Research in the field of education has demonstrated that students with a proactive personality exhibit a greater propensity to engage with academic tasks, persist through challenges, and actively seek resources to achieve educational success (Wang et al., 2013; Johari et al., 2022; Zimmermann et al., 2024), ultimately leading to superior academic performance. A study by Dong and Liu (2016) focusing on university students revealed that individuals with high levels of initiative display more active learning behaviors and greater engagement in the learning process. Such individuals are characterized by heightened autonomous motivation (Gao et al., 2015) and intrinsic motivation (Chen and Kao, 2014), as well as increased enthusiasm and resilience, enabling them to seize opportunities, exert greater effort, and proactively adapt to their environment (Liu et al., 2020). These behaviors are positively correlated with academic achievement, underscoring the role of proactive students in taking greater ownership of their learning and academic outcomes (Parker et al., 2010).

Proactive individuals exhibit a pronounced tendency to engage in goal-directed behaviors and demonstrate a superior capacity to anticipate and address challenges within their learning environments. Empirical evidence from Ma et al. (2024) highlights that proactive students display heightened persistence, improved time management, and advanced problem-solving skills, all of which are positively associated with academic success. Moreover, such individuals are more inclined to establish long-term academic objectives and implement strategic actions to achieve them, further reinforcing the correlation between proactive traits and enhanced academic performance. Based on these findings, we propose the following hypothesis 1.

H1: Proactive personality plays a role in promoting students' academic achievement.

### 2.2 The mediating role of academic self-efficacy

Drawing on the Job Demands-Resources (JD-R) theory, academic self-efficacy—defined as students' confidence in their capacity to execute and excel in academic tasks—represents a pivotal psychological resource that significantly shapes motivation, persistence, and learning strategies, all of which are critical determinants of academic achievement. Rooted in the theory of self-efficacy, initially articulated by Bandura in the 1970s, this construct underscores the profound influence of individuals' beliefs in their capabilities on their actions, emotional responses, and motivational drive, particularly when confronted with challenges (Bandura, 1978). Within the academic domain, self-efficacy operates as a fundamental driver of academic behavior, modulating core cognitive and motivational processes such as goal-setting, self-regulation, and task engagement (Pintrich and de Groot, 1990). As a key individual resource within the JD-R framework, academic self-efficacy has been empirically established as a robust predictor of achievement (Bandura, 1997). Students endowed with heightened self-efficacy are more inclined to adopt adaptive learning behaviors, establish ambitious academic objectives, and demonstrate resilience in overcoming obstacles, thereby fostering superior academic outcomes (Zimmerman, 2000; Schunk and DiBenedetto, 2020).

Proactive personality, as conceptualized in the literature, is characterized by its capacity to initiate positive motivational processes, such as enhanced self-efficacy, which subsequently facilitates improved learning outcomes. Parker et al. (2010) advanced a model of proactive motivational processing, positing that both contextual factors—including interpersonal dynamics, peer support, and work-related pressures—and individual differences—such as proactive personality, self-regulation, and emotional control—shape the proactive motivational state. This state, encompassing self-efficacy, intrinsic motivation, and integrated motivation, in turn influences proactive goal pursuit and ultimate performance outcomes. From a JD-R perspective, proactive personality serves as a critical individual resource that interacts with work resources (e.g., social support, learning opportunities) to buffer academic demands and enhance academic performance. Empirical studies have further identified academic self-efficacy as a critical mediator in the relationship between proactive personality and academic achievement (Chen et al., 2021). Specifically, proactive personality fosters the development of academic self-efficacy through goal-setting, feedback-seeking, and self-directed learning behaviors (Lin et al., 2014). Enhanced academic self-efficacy, in turn, amplifies students' motivation, persistence, and adoption of effective learning strategies, thereby directly enhancing academic performance (Saks, 2024). Proactive students exhibit a stronger belief in their academic capabilities, which facilitates deeper engagement in learning and superior academic outcomes (Fu et al., 2024). Proactive personality has been shown to positively predict academic performance, with this relationship significantly mediated by academic self-efficacy. Students with high levels of proactive personality and academic self-efficacy are more likely to engage in behaviors such as seeking assistance, maintaining organizational skills, and sustaining motivation, all of which contribute to academic success (Schunk et al., 2010). Furthermore, proactive students with elevated academic self-efficacy are more inclined to employ adaptive learning strategies, including effective time management, active note-taking, and deep learning approaches, which further bolster academic performance (Schunk and DiBenedetto, 2020). By cultivating a robust belief in their abilities, academic self-efficacy enables proactive students to effectively translate their initiative into measurable academic achievements. Based on these findings and the JD-R framework, we propose Hypothesis 2.

H2: Academic self-efficacy mediates the relationship between proactive personality and students' academic achievement.

### 2.3 The moderating role of perceived social support

Based on the Job Demands-Resources (JD-R) theory, social support constitutes a critical work resource, encompassing feedback and resources provided by university staff, peers, and other groups, which students perceive as accessible and utilizable (Ye et al., 2021). This support extends beyond formal and informal relationships within the academic community—including peers, lecturers, and professors—to serve as a vital source of assistance and encouragement in students' academic development (Mishra, 2020). Concurrently, proactive personality and academic self-efficacy, as fundamental individual resources, play a pivotal role in fostering personal transformation and self-motivation, enabling students to engage more profoundly in learning and to effectively navigate academic challenges (Seibert et al., 1999; Bandura, 1997).

As a significant work resource, social support not only facilitates personal growth and the attainment of organizational goals but also bolsters students' ability to manage academic pressures by reinforcing their sense of value and encouragement, thereby enhancing academic outcomes (Cohen and Wills, 1985). Crucially, the impact of social support hinges on its perception and internalization by students, a process termed “perceived social support” (Zimet et al., 1988). Perceived social support influences mental health and emotional regulation while also serving as a key determinant of academic self-efficacy (Zimet et al., 1988). Empirical evidence indicates that perceived social support aids students in managing stress, elevating self-esteem, and promoting mental wellbeing, all of which are integral to academic success (Rosenfeld et al., 1998).

For students, support from family, peers, and teachers can significantly bolster academic self-efficacy, particularly when confronting academic challenges. Such support mitigates self-doubt and reinforces confidence in achieving academic success (Bandura, 1997). Consequently, perceived social support might serve as a moderating factor in the relationship between academic self-efficacy and academic achievement. Specifically, social support functions as a safety net that can amplify the positive effects of self-efficacy on academic outcomes or buffer against the detrimental effects of low self-efficacy (Schunk and DiBenedetto, 2020). By alleviating the anxiety and stress associated with academic challenges, social support enhances resilience in students with high self-efficacy, enabling them to persist in pursuing their academic goals despite difficulties (Cohen and Wills, 1985). For instance, Soria and Stebleton (2012) found that perceived social support from family and friends improved the ability of students with low academic self-efficacy to manage academic stress, resulting in better academic outcomes. Additionally, research indicates that perceived social support positively influences motivation, which in turn enhances academic achievement (Wentzel, 1998). Schunk and DiBenedetto (2020) further demonstrated that support from teachers and peers strengthened students' belief in their academic capabilities, particularly when their self-efficacy was initially low. In other words, students who perceived strong support from significant others were more likely to persist in academic tasks and employ adaptive learning strategies, ultimately leading to improved academic performance (Zimmerman, 2000). This underscores the dual role of perceived social support as both a buffer against adversity and a motivator, reinforcing the link between academic self-efficacy and achievement. Conversely, in the absence of social support, the impact of academic self-efficacy on achievement may diminish, as students may experience feelings of isolation and helplessness, thereby undermining academic performance (Malecki and Demaray, 2003). Based on these insights, we propose Hypothesis 3.

H3: Academic self-efficacy mediates the relationship between proactive personality and students' academic achievement.

**Figure 1:**
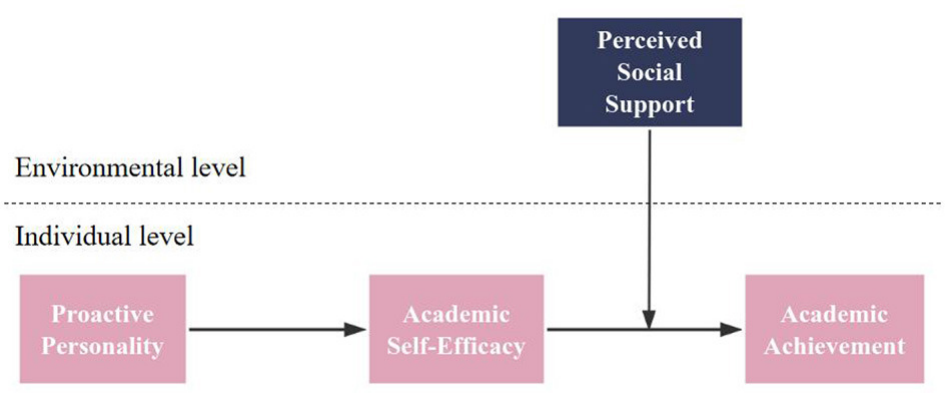
Proposed theoretical framework of the study.

In summary, based on the hypothesized relationships of the core variables, this study constructs a cross-level moderated mediation model (see Figure 1), in which proactive personality influences students' academic achievement through academic self-efficacy, while perceived social support moderates this indirect relationship.

## 3 Methods

### 3.1 Participants

#### 3.1.1 Ethical considerations

This study received ethical approval from the Ethics Committee of North China University of Science and Technology (approval number 20250115). Prior to participation, informed consent was obtained from all participants, who were informed of their right to withdraw from the study at any time without consequence. To protect participant confidentiality, all personally identifiable information was anonymized.

#### 3.1.2 Data collection

Data collection was conducted using WJX, a professional online survey platform widely utilized in China, serving over 90% of higher education institutions in the country. Participants were recruited from universities via convenience sampling. Approximately 1,200 questionnaires were distributed through the WJX platform over a 1-week period. Of the 1,153 responses received, 1,102 met the inclusion criteria, yielding an effective response rate of 95.6%. The sample comprised 444 males (40.3%) and 658 females (59.7%). By academic year, participants included 240 freshmen (21.8%), 252 sophomores (22.9%), 230 juniors (20.9%), 224 seniors (20.3%), and 156 fifth-year students (14.1%). Additionally, 133 participants (12.1%) held class officer positions, while 969 (87.9%) did not. Participant demographic characteristics are detailed in Supplementary Table 1. The data supporting this study are not publicly available but can be accessed from the corresponding author upon reasonable request.

### 3.2 Measures

The survey questionnaire for this study comprised five sections: demographic information (gender, age, education, experience), predictor variable (Proactive Personality, PP), moderator variable (Perceived Social Support, PSS), mediator variable (Academic Self-Efficacy, ASE), and criterion variable (Academic Achievement, AA). All constructs in the conceptual model (Figure 1) were measured using validated scales from established literature, which have been widely utilized in prior research. Items were rated on a 7-point Likert scale (1 = strongly disagree, 7 = strongly agree). Data were analyzed using SPSS version 22.0 and Mplus version 8.3, with statistical techniques including correlation analysis, principal component analysis, *t*-tests, and parametric statistics.

#### 3.2.1 Proactive personality scale (PPS)

The proactive personality traits in this study were evaluated using a scale originally developed by Bateman and Crant (1993) and subsequently adapted by Shang and Gan (2009). The scale comprises 11 items, including statements such as, “If I see someone in trouble, I help out in any way I can.” Higher scores on this scale reflect greater levels of proactive personality characteristics. The internal consistency of the scale was measured using Cronbach's alpha coefficient, which yielded a value of 0.949 in this study, indicating excellent reliability.

#### 3.2.2 Academic achievement scale (AAS)

The academic achievement of students was assessed using the academic achievement Scale, originally developed by Bao and Zhang (2012). This scale measures academic performance across three dimensions: professional literacy, core competencies, and academic outcomes. Comprising ten items, the scale includes statements such as “My understanding of the cutting-edge developments in my field” and “My mastery of the fundamental theoretical knowledge in my field,” which align closely with the criteria commonly employed by employers in evaluating potential candidates. Higher scores on the scale correspond to greater academic achievements during the students' university tenure, providing a comprehensive reflection of their overall academic performance. In this study, the scale demonstrated strong internal consistency, with a Cronbach's alpha coefficient of 0.946, indicating high reliability.

#### 3.2.3 Academic self-efficacy scale (ASES)

The Academic Self-Efficacy Scale, originally developed by Pintrich and de Groot (1990) and subsequently adapted by Liang (2000), was employed to assess the academic self-efficacy levels of university students. The scale comprises 22 items, organized into two dimensions: academic ability self-efficacy and academic behavior self-efficacy, each containing 11 items. Items 14, 16, and 17 are reverse-scored. In this study, Cronbach's alpha coefficients for academic ability self-efficacy and academic behavior self-efficacy were 0.946 and 0.945, respectively, indicating excellent internal consistency for both dimensions.

#### 3.2.4 Perceived social support scale (PSSS)

The Perceived Social Support Scale (PSSS), originally developed by Zimet et al. (1990) and subsequently translated and adapted by Jiang (1999), was utilized in this study. The scale comprises 12 items, organized into three dimensions: family support, friend support, and other support. In this study, the scale demonstrated a high level of internal consistency, with Cronbach's alpha coefficient of 0.947, indicating robust reliability.

## 4 Results

### 4.1 Reliability and validity of the model

Table 1 presents the values of Cronbach's alpha, composite reliability (CR), average variance extracted (AVE), and factor loadings, which were computed to evaluate the reliability and validity of the model. CR, which measures the internal consistency among variables, exceeded the minimum threshold of 0.700, as recommended by Nunally and Bernstein (1978). Factor loadings, used to assess the reliability of individual items, also met the minimum acceptance criterion of 0.700. Cronbach's alpha values, indicative of internal consistency reliability, surpassed the threshold of 0.700, further supporting the model's reliability (Nunally and Bernstein, 1978). Additionally, the average variance extracted (AVE) values for the constructs reflect the proportion of variance captured by each construct relative to the variance attributable to measurement error. In this study, the AVE values for all constructs exceeded the minimum acceptable threshold of 0.500, as established by Bagozzi and Yi (1988), thereby confirming the constructs' convergent validity.

**Table 1:** Reliability, convergent and discriminant validity.

**Proactive personality**		**VIF**	**Loadings**
CR = 0.949 AVE = 0.627 Alpha = 0.949	PP_01	2.521	0.796
	PP_02	2.524	0.798
	PP_03	2.418	0.785
	PP_04	2.580	0.799
	PP_05	2.383	0.780
	PP_06	2.533	0.796
	PP_07	2.567	0.791
	PP_08	2.432	0.788
	PP_09	2.496	0.791
	PP_10	2.508	0.794
	PP_11	2.442	0.789
**Academic self-efficacy**		**VIF**	**Loadings**
Learning ability self-efficacy	ASE_01	2.384	0.781
CR = 0.946 AVE = 0.614 Alpha = 0.946	ASE_02	2.382	0.782
	ASE_03	2.365	0.781
	ASE_04	2.314	0.799
	ASE_05	2.382	0.779
	ASE_06	2.598	0.804
	ASE_07	2.439	0.786
	ASE_08	2.274	0.765
	ASE_09	2.495	0.793
	ASE_10	2.644	0.808
	ASE_11	2.277	0.768
Learning behavioral self-efficacy	ASE_12	2.322	0.775
CR = 0.945 AVE = 0.611 Alpha = 0.945	ASE_13	2.466	0.789
	ASE_14	2.390	0.784
	ASE_15	2.308	0.772
	ASE_16	2.431	0.788
	ASE_17	2.296	0.773
	ASE_18	2.474	0.792
	ASE_19	2.343	0.774
	ASE_20	2.501	0.796
	ASE_21	2.256	0.767
	ASE_22	2.469	0.791
**Perceived social support**		**VIF**	**Loadings**
CR = 0.947 AVE = 0.598 Alpha = 0.947	PSS_01	2.292	0.700
	PSS_02	2.311	0.773
	PSS_03	2.491	0.793
	PSS_04	2.417	0.784
	PSS_05	2.401	0.780
	PSS_06	2.276	0.761
	PSS_07	2.370	0.780
	PSS_08	2.305	0.769
	PSS_09	2.161	0.750
	PSS_10	2.275	0.767
	PSS_11	2.406	0.784
	PSS_12	2.304	0.771
**Academic achievement**		**VIF**	**Loadings**
CR = 0.946 AVE = 0.638 Alpha = 0.946	AA_01	2.429	0.788
	AA_02	2.574	0.801
	AA_03	2.582	0.805
	AA_04	2.595	0.806
	AA_05	2.466	0.792
	AA_06	2.431	0.790
	AA_07	2.522	0.799
	AA_08	2.621	0.809
	AA_09	2.411	0.786
	AA_10	2.647	0.810

To assess the potential for common method bias (CMB), multiple diagnostic approaches were employed, as recommended in prior literature. First, variance inflation factor (VIF) values for all items across study variables were calculated, with results indicating no evidence of CMB, as all VIF values were below the threshold of 5 (Kock, 2015; see Table 1). Second, Harman's single-factor test (Harman, 1967) was conducted, revealing that the cumulative variance explained by a single factor was 26.527%, well below the critical threshold of 40%, further supporting the absence of CMB. Finally, in line with the criteria proposed by Bagozzi et al. (1991), inter-variable correlation coefficients were examined, with all values remaining below 0.900, confirming that common method bias does not pose a significant concern in this study.

### 4.2 Measurement model

Confirmatory factor analysis (CFA) was conducted using SPSSAU to assess the model's fit. As shown in Table 2, the Chi-square to degrees of freedom ratio (χ^2^/*df* ) was 1.023, which falls below the threshold of 3, indicating an acceptable fit as per established guidelines (Hu and Bentler, 1999). Additionally, the model demonstrated strong fit indices, including a Goodness of Fit Index (GFI) of 0.951, an Adjusted Goodness of Fit Index (AGFI) of 0.952, a Comparative Fit Index (CFI) of 0.999, a Tucker-Lewis Index (TLI) of 0.999, and a Normed Fit Index (NFI) of 0.967. Furthermore, the Root Mean Residual (RMR) was 0.066, and the Root Mean Square Error of Approximation (RMSEA) was 0.005, both of which are within the recommended thresholds for model fitness (Hair et al., 2010; Hu and Bentler, 1999). Collectively, these results confirm the robustness of the model's fit to the data.

**Table 2:** Measurement model.

**Acceptable range**	**Fitness criteria**	**Measurement model**
< 3	Chisq/df	1.023
>0.90	GFI	0.951
>0.9	AGFI	0.952
>0.9	CFI	0.999
>0.9	TLI	0.999
>0.9	NFI	0.967
< 0.09	RMR	0.066
< 0.10	RMSEA	0.005

### 4.3 Descriptive statistics

Table 3 shows the mean, Standard deviation, and correlation values, where all variables are positively and significantly correlated at a significance value of 0.01.

**Table 3:** Descriptive statistics and correlations.

**Variables**		**Mean**	**S.D**.	**1**	**2**	**3**	**4**
1	PP	4.916	1.490		0.361^**^	0.304^**^	0.347^**^
2	ASE	5.038	1.058			0.272^**^	0.366^**^
3	PSS	5.055	1.414				0.297^**^
4	AA	4.875	1.516				

^**^ *p* < 0.01.

### 4.4 Hypotheses testing

**Figure 2:**
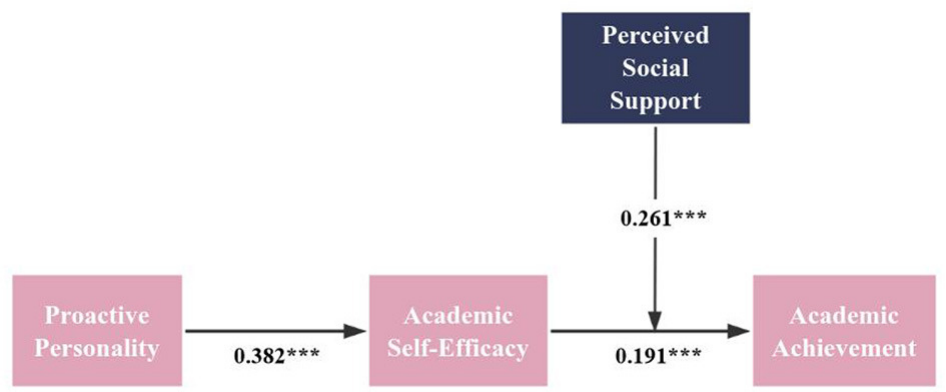
Structural model. ***p < 0.001.

Table 4 presents the results derived from a bootstrapping analysis with 5,000 samples, following the methodology recommended by Hayes (2015, 2018). The direct effect analysis reveals a significant positive influence of proactive personality (PP) on academic achievement (AA) (*b* = 0.252, SE = 0.030, *t* = 8.482, *p* < 0.001, 95% CI [0.193, 0.310]), thereby supporting Hypothesis 1. The path coefficients in Figure 2 show that PP was positively related to academic self-efficacy (ASE) (*b* = 0.382, *p* < 0.001), academic self-efficacy (ASE) was positively related to AA (*b* = 0.191, *p* < 0.001), which indicates that ASE mediates the influence of PP on AA. Additionally, the indirect effect analysis of Table 4 also demonstrates that ASE significantly mediates the relationship between PP and AA (*b* = 0.102, SE = 0.013, 95% CI [0.078, 0.125]), confirming Hypothesis 2.

**Table 4:** Hypotheses testing.

**Models**	**Effect**	**SE**	***t***	***p***	**LL**	**UL**
**Direct effect and indirect effects**						
Direct effect	0.252	0.030	8.482	0.000	0.193	0.310
Indirect effect	0.102	0.013	8.418	0.000	0.078	0.125
**Interaction effects**						
ASE → AA	0.348	0.042	8.372	0.000	0.267	0.430
PSS → AA	0.162	0.031	5.243	0.000	0.101	0.222
ASE × PSS → AA	−0.109	0.027	−3.999	0.000	−0.162	−0.055
**Conditional indirect effects**						
Less than mean	0.129	0.017	12.297	0.001	0.097	0.164
At mean	0.089	0.013	8.454	0.000	0.065	0.114
Above than mean	0.050	0.016	8.129	0.001	0.019	0.082
**Moderated mediation index**						
PP → ASE × PSS → AA	−0.028	0.008			−0.043	−0.013

**Figure 3:**
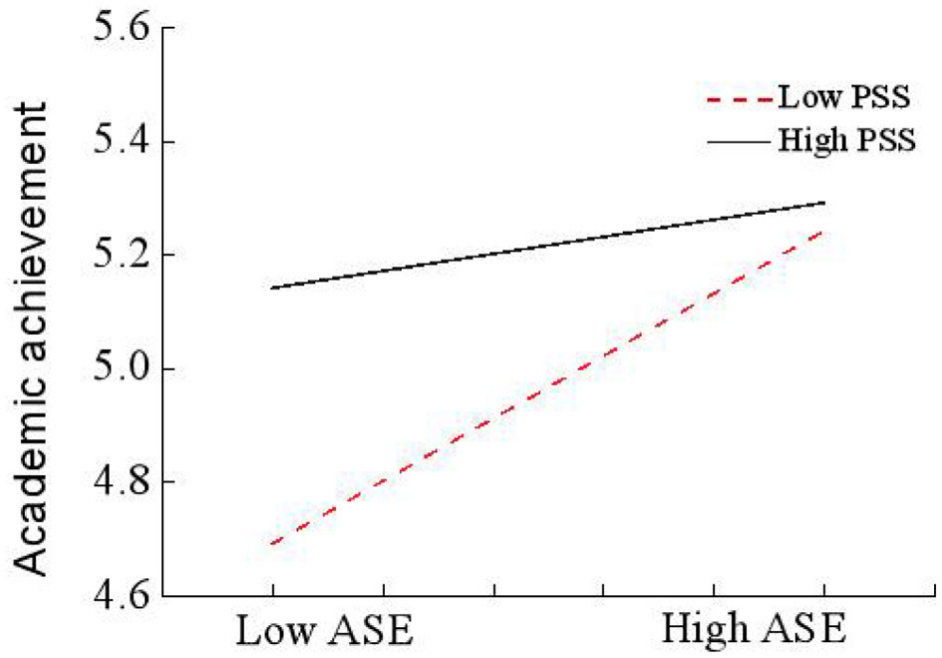
The interaction between academic self-efficacy (ASE) and perceived social support (PSS) on academic achievement (AA).

The interaction effects, as shown in the second portion of Table 4, indicate that ASE is positively associated with AA (*b* = 0.348, SE = 0.042, *t* = 8.372, *p* < 0.001, 95% CI [0.267, 0.430]). Similarly, perceived social support (PSS) exhibits a significant positive relationship with AA (*b* = 0.162, SE = 0.031, *t* = 5.243, *p* < 0.001, 95% CI [0.101, 0.222]). Notably, the interaction term (ASE × PSS) also significantly influences AA (*b* = −0.109, SE = 0.027, *t* = −3.999, *p* < 0.001, 95% CI [−0.162, −0.055]). Figure 2 also shows that the interaction between ASE and PSS significantly predicted AA (*b* = 0.261, *p* < 0.001), providing support for Hypothesis 3. To further elucidate the moderation effect, an interaction plot was constructed at standard deviation (SD) levels, the interaction effect (*b* = −0.109) is negative, which means that the positive effect of ASE on AA is weaker at higher levels of PSS (Figure 3).

The conditional indirect effects and moderated mediation index, presented in the third and fourth portions of Table 4, reveal that PSS moderates the indirect relationship between PP and AA via ASE. Specifically, the indirect effect is stronger at lower levels of PSS (*b* = 0.129, SE = 0.017, *t* = 12.297, *p* < 0.01, 95% CI [0.097, 0.164]), moderate at mean levels of PSS (*b* = 0.089, SE = 0.013, *t* = 8.454, *p* < 0.001, 95% CI [0.065, 0.114]), and weaker at higher levels of PSS (*b* = 0.050, SE = 0.016, *t* = 8.129, *p* < 0.01, 95% CI [0.019, 0.082]). Furthermore, the moderated mediation index indicates that the indirect effect is strongest at low PSS and weakest at high PSS, higher PSS reduces the strength of the ASE → AA relationship (*b* = −0.028, SE = 0.008, 95% CI [−0.043, −0.013]), thereby substantiating Hypothesis 4.

## 5 Discussion

This study investigates the relationship between proactive personality (PP) and academic achievement (AA) among Chinese university students, employing the Job Demands-Resources (JD-R) theory to explore the mediating role of academic self-efficacy (ASE) and the moderating influence of perceived social support (PSS). The research seeks to elucidate the mechanisms through which PP influences AA in contemporary educational contexts. Correlational analyses demonstrate that PP significantly and positively predicts AA, indicating that students with higher levels of PP are more likely to engage in goal-setting, proactive coping strategies, and optimization of learning processes. Such behaviors include seeking academic resources and formulating structured study plans, which collectively contribute to enhanced academic performance. These findings are consistent with prior research in organizational settings, which has established that proactive individuals exhibit superior job performance (Zhu et al., 2017; Wei et al., 2021). The results underscore the generalizability of proactive behavior as a predictor of success across both workplace and academic environments, suggesting that individuals who exhibit initiative, adaptability, and a proactive approach to goal attainment are more likely to achieve favorable outcomes. This aligns with international studies (Kirby et al., 2002), which have identified a positive correlation between proactivity and individual performance in learning contexts. As a critical personal resource, PP enables students to effectively navigate academic demands, mitigate stress, and optimize their academic achievements.

The study further reveals that proactive personality (PP) exerts a significant and positive influence on academic self-efficacy (ASE), which, in turn, significantly and positively predicts academic achievement (AA). These findings indicate that ASE fully mediates the relationship between PP and AA, thereby confirming the study' s hypothesis and validating the applicability of the proactive motivation and driving model (Parker et al., 2010) within the educational domain. Specifically, PP enhances ASE, which subsequently drives academic outcomes. This mechanism can be attributed to the tendency of university students with a proactive disposition to exhibit greater confidence in their capacity to accomplish academic tasks through self-directed efforts (Ma et al., 2024). By actively cultivating a supportive learning environment, these students bolster their academic performance (Wang et al., 2021). The results also suggest that individuals with a stronger proactive disposition possess heightened confidence in both their abilities and their surroundings. Regardless of external challenges, they maintain a belief in their capacity to adapt and succeed, thereby demonstrating elevated levels of ASE (Zhu et al., 2017). This confidence further translates into sustained academic effort, resilience in the face of adversity, and ultimately, superior academic outcomes.

From a resource-based perspective, this study corroborates that environmental resources, such as social support, amplify the impact of individual resources, enabling individuals to more effectively navigate demands (Bakker and Demerouti, 2007). Perceived social support (PSS) moderates the relationship between academic self-efficacy (ASE) and academic achievement (AA), such that the positive predictive effect of ASE on AA is significantly enhanced in the presence of higher levels of social support from family, peers, or educators. This underscores the role of social support as a pivotal environmental resource, providing students with emotional, informational, and instrumental assistance, which in turn elevates their confidence and learning motivation (Cohen and Wills, 1985). In contexts characterized by robust social support, students with a proactive personality are more likely to perceive external encouragement and reinforcement, thereby further bolstering their ASE and more effectively channeling their proactive tendencies into academic success (Wang et al., 2024). These findings are consistent with the Job Demands-Resources (JD-R) Theory, which highlights the synergistic interplay between environmental and individual resources in shaping outcomes.

### 5.1 Theoretical implications

This study enriches the research field on the relationship between proactive personality and academic achievement by uncovering the mediating mechanism of academic self-efficacy and the moderating role of perceived social support. The findings support Social Cognitive Theory (Bandura and National Institute of Mental Health, 1986), highlighting the interplay between personal factors (proactive personality), environmental factors (perceived social support), and cognitive factors (academic self-efficacy) in shaping students' academic performance. Additionally, this study provides empirical support for the application of the Job Demands-Resources Theory (Bakker and Demerouti, 2007) in the educational context, demonstrating that individual resources (proactive personality) and environmental resources (social support) jointly influence students' academic outcomes.

### 5.2 Practical implications

This study holds significant implications for educational practice. First, schools and teachers should prioritize fostering proactive personality traits in students by encouraging goal-setting, active participation in classroom activities, and self-directed learning. These practices help students accumulate successful experiences, thereby enhancing their academic self-efficacy. Second, teachers and parents should pay attention to students' perceptions of social support by providing emotional support, academic guidance, and instrumental assistance to create a supportive learning environment. Empirical research showed that by systematically constructing supportive teacher-student interaction relationships, students' academic self-efficacy can be significantly enhanced (Chen et al., 2021). Finally, educators should focus on improving students' academic self-efficacy by breaking down learning tasks, providing role models of success, and teaching effective learning strategies, all of which help students develop confidence in their abilities.

### 5.3 Limitations and implications for future research

This study has several limitations. First, the use of a cross-sectional design precludes the establishment of causal relationships among the variables. Future research could employ longitudinal or experimental designs to further validate the dynamic relationships between proactive personality, academic self-efficacy, and perceived social support. Second, Harman's test is considered a weak test for CMB in recent literature, the reliance on self-reported data may introduce common method bias. Future studies could incorporate multi-source data, such as teacher evaluations and parent reports, to enhance the reliability of the findings. Finally, this study examined perceived social support as a holistic construct. Future research could explore the specific roles of different sources of social support (e.g., family, friends, and teachers) in the relationship between proactive personality and academic achievement. Harman's test is considered a weak test for CMB in recent literature.

## 6 Conclusions

Grounding itself in the Job Demands-Resources Theory, this study elucidates the mechanisms through which proactive personality influences students' academic achievement, highlighting the mediating role of academic self-efficacy and the moderating role of perceived social support. The findings indicate that proactive personality, as a crucial individual resource, directly and indirectly enhances academic performance, while perceived social support, as an environmental resource, amplifies the effects of proactive personality. These insights provide a novel perspective for understanding individual differences in students' academic achievement and offer valuable guidance for educational practice. By fostering proactive personality traits, enhancing academic self-efficacy, and cultivating a supportive social environment, educators and parents can help students better navigate academic demands and achieve academic success.

## Data Availability

The raw data supporting the conclusions of this article will be made available by the authors, without undue reservation.
