# Supplementary material for: Impacts of proactive personality on students' academic achievement: a moderated mediation model
Source: Front Psychol. 2025 Jun 9;16:1596032. doi: 10.3389/fpsyg.2025.1596032 (PMC12183227; doi:10.3389/fpsyg.2025.1596032)
Supplement: Supplementary file 1 [file Table_1.DOCX]

Supplementary Material

# Table 1 Participant demographic characteristics

| **Characteristic** | **n (%)** |
| --- | --- |
| **Total** | 1102(100) |
| **Gender** |  |
| Male | 444(40.3) |
| Female | 658(59.7) |
| **Academic year** |  |
| freshmen | 240(21.8) |
| sophomores | 252(22.9) |
| juniors | 230(20.9) |
| seniors | 224(20.3) |
| fifth-year | 156(14.1) |
| **Leadership role** |  |
| Class officer | 133(12.1) |
| Non-officer | 969(87.9) |
